# Fracture Detection in Traumatic Pelvic CT Images

**DOI:** 10.1155/2012/327198

**Published:** 2012-01-04

**Authors:** Jie Wu, Pavani Davuluri, Kevin R. Ward, Charles Cockrell, Rosalyn Hobson, Kayvan Najarian

**Affiliations:** ^1^Department of Computer Science, Virginia Commonwealth University, 401 West Main Street, Richmond, VA 23284, USA; ^2^Department of Electrical and Computer Engineering, Virginia Commonwealth University, 401 West Main Street, Richmond, VA 23284, USA; ^3^Department of Emergency Medicine, Virginia Commonwealth University, 401 West Main Street, Richmond, VA 23284, USA; ^4^Virginia Commonwealth University Reanimation Engineering Science Center (VCURES), Virginia Commonwealth University, 401 West Main Street, Richmond, VA 23284, USA; ^5^Department of Radiology, Virginia Commonwealth University, 401 West Main Street, Richmond, VA 23284, USA

## Abstract

Fracture detection in pelvic bones is vital for patient diagnostic decisions and treatment planning in traumatic pelvic injuries. Manual detection of bone fracture from computed tomography (CT) images is very challenging due to low resolution of the images and the complex pelvic structures. Automated fracture detection from segmented bones can significantly help physicians analyze pelvic CT images and detect the severity of injuries in a very short period. This paper presents an automated hierarchical algorithm for bone fracture detection in pelvic CT scans using adaptive windowing, boundary tracing, and wavelet transform while incorporating anatomical information. Fracture detection is performed on the basis of the results of prior pelvic bone segmentation via our registered active shape model (RASM). The results are promising and show that the method is capable of detecting fractures accurately.

## 1. Introduction

Pelvic fractures are high energy injuries that constitute a major cause of death in trauma patients. According to the Centers for Disease Control and Prevention (CDC), trauma injury kills more people between the ages of 1 and 44 than any other disease or illness. Among different types of trauma with a high impact on the lives of Americans, traumatic pelvic injuries, caused mainly by sports, falls, and motor vehicle accidents, contribute to a large number of mortalities every year [1, 2]. Traumatic pelvic injuries and associated complications, such as severe hemorrhage multiple organ dysfunction syndrome (MODS), result in the mortality rate from 8.6% to 50% [3]. When combined with other injuries in the body, for instance, the abdomen, the chance of mortality is even higher [4]. In general, a pelvic fracture can be associated hemorrhage, neurologic injury, vascular injury, and organ damage, as all of the vital structures run through pelvis. Pain and impaired mobility are normally the results of nerve and internal organ damage associated with the pelvic fracture [5–7].

Patient data, in particular, medical images such as computed tomography (CT) images, contain a significant amount of information, and it is crucial for physicians to make diagnostic decisions as well as treatment planning on the basis of this information and other patients' data. Currently, a large portion of the data is not optimally and comprehensively utilized, because information held in the data is inaccessible through visual observation or simple traditional computational methods. Information contained in pelvic CT images is a very important resource for the assessment of the severity and prognosis of the injuries. Each pelvic CT scan consists of several slices; each slice contains a large amount of data that may not be thoroughly and accurately analyzed via visual inspection. In addition, in the field of trauma, physicians frequently need to make quick decisions based on large amount of information. Hence, a computer-assisted pelvic trauma decision-making system is crucial and necessary for assisting physicians in making accurate diagnostic decisions and determining treatment planning in a short period.

Automated fracture detection from segmented bones in traumatic pelvic injuries can help physicians examine the pelvic CT images and to detect the injury severity within a short period. Extraction of features such as presence and location of fracture, hemorrhage, and displacement between the fractured bones in an automated fashion is vital for such injuries. Identification of fracture alone is not sufficient to assess the injury severity. Therefore, details of the fracture such as distance and angle between the fractured bones must be taken into account. However, the task of pelvic bone segmentation and fracture detection is very challenging due to low resolution of CT images, complex pelvic structures, variations in bone shape, and size from patient to patient. Adding to these complexities, the presence of noise, partial volume effects, and in-homogeneities in the CT images make the task of fracture detection very challenging. The objective of this study is to design a computer-assisted system that helps radiologists better and further assess the bone fractures in pelvic region. It also illustrates the fracture bones in a clearer and more visible manner. In particular, mild and small fractures, while still partially visible in the CT images, are sometimes considered as “irregularities” that need further investigation by the radiologists in the first read, as radiologists may not be able to reliably label them as fractures due to the quality of the CT as well as the volume of the data to be processed. For these situations, it normally takes multiple reads to identify and determine the confirmation on the existence and/or details of fracture. A machine-based analysis can consider and process detailed information from several neighboring slices to provide radiologists with clues as to whether one particular slice contains a fracture and if so extract details such as the separation among the pieces.

While there have been few studies directly focusing on fracture detection in pelvic CT images, there are many closely related work. Moghari and Abolmaesumi [8] utilized a global registration method for multifragment fracture fixation in femur bone. However, the method suffers from initial alignment errors, and the dataset includes only femur bone generated randomly from 3D data points. Moghari and Abolmaesumi [9] proposed a technique to automatically register multiple bone fragments of a fracture using a global registration method guided by a statistical anatomical atlas model. Due to the limited number of bone models, the method is unable to capture all variations of femur. Winkelbach et al. [10] presented an which is approach based on a modified version of Hough Transformation and registration techniques for estimating the relative transformations between fragments of a broken cylindrical structure. This method is designed for computer-aided bone alignment, such as fractured long bones and fracture reduction in surgery. However, the approach is not fully automatic and requires a significant amount of human supervision. Another work, by Ryder et al. [11] explored using nonvisual methods to detect fractures. In addition, there are image processing methods for fracture detection applies to X-ray images [12–14]. Douglas et al. [12] focused on early detection of fractures with low-dose digital X-ray images in a pediatric trauma unit. Tian et al. [13] determined the presence of femoral fracture by measuring the neck-shaft angle of the femur. Lum et al. [14] used three-texture features combined with a classifier to detect radius and femur fractures. This method may suffer from the imbalanced dataset. The majority of these X-ray image processing methods may not be applicable to fracture detection in CT images because of the variation in image intensities and resolution between X-ray and CT images.

Even though few studies have been conducted on fracture detection from pelvic CT scans, several segmentation techniques have been created for medical images of various regions of human body, that is, brain, abdomen, and so forth. These methods include threshold-based techniques, region growing, classifiers, clustering, Markov random field models, artificial neural networks, deformable models, atlas-guided methods, knowledge-based methods. Thresholding techniques segment an image by creating a binary partition on the basis of the image intensities [15]. The drawback is that they cannot be effectively applied to multichannel images. The deformable model approaches start with the initial contour placement near the desired boundary, and then, the contour is improved through an iterative relaxation process [16–18]. The disadvantage is that these methods require manual interaction for the selection of initial position and appropriate parameters of the model. Atlas-guided methods utilize a standard atlas or template for segmentation [19]. The atlas used as the reference frame is generated on the basis of the previously known anatomical information. However, due to anatomical variability across individuals, accurate segmentation of complex structures remains as a challenging task. Clustering algorithms, also referred to as unsupervised methods [20, 21], while successful in some applications, they can be sensitive to noise and variations in intensity. In addition, the calculation can become computationally expensive when the clusters have a large number of pixels.

This study develops an automated hierarchical algorithm to detect fracture in pelvic bones using a hierarchical method combining several of the above-motioned methods in different steps. Fracture detection is performed using the proposed automated segmentation method, called registered active shape model (RASM), along with wavelet transformation, adaptive windowing, boundary tracing, and masking.

The rest of the paper is organized as follows.  Section 2 provides the methods used for pelvic bone segmentation and fracture detection.  Section 3 includes the results obtained using the proposed methods and discusses the obtained results.  Section 4 concludes the proposed methods and provides the future work of the study.

## 2. Methods

**Figure 1:**
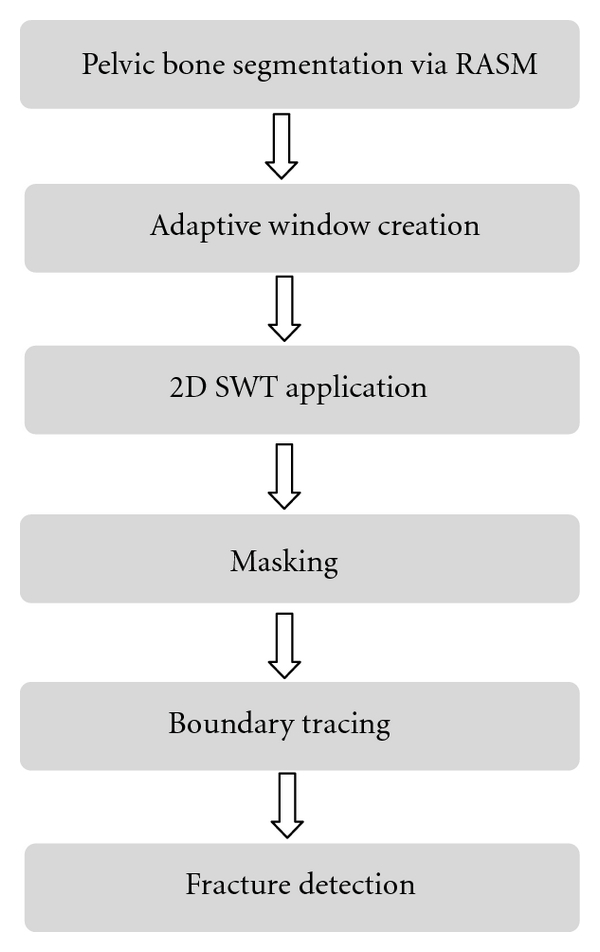
Schematic diagram of pelvic bone fracture detection.

Automated fracture detection is important for making fast and accurate decisions and treatment planning. In order to successfully detect pelvic bone fractures, utilizing the bone information contained in pelvic CT images is crucial.  Figure 1 illustrates the overall process of the proposed automated fracture detection. The proposed fracture detection method involves automated bone segmentation using registered active shape model (RASM), adaptive windowing, 2D stationary wavelet transform, masking, and boundary tracing. Each step in the process is explained in detail in the following subsections.

### 2.1. Multilevel Segmentation of Bone in Pelvic CT Scans

**Figure 2:**
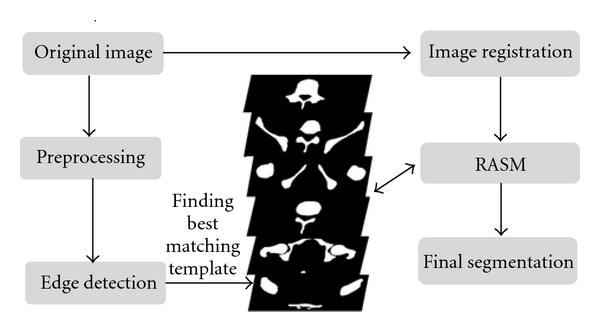
Schematic diagram of pelvic bone segmentation.

Segmentation is a vital step in analyzing pelvic bones in CT images and the first step in fracture detection. Specifically, bone segmentation helps extract the bones from the images that are later used for detecting fractures. Our previous work was focused on the segmentation of pelvic bones in CT scans [22]. In this paper, a new segmentation algorithm for multilevel pelvic CT scans was developed. This is shown in Figure 2. This new segmentation technique consists of four main parts: preprocessing, edge detection, shape matching and Registered Active Shape Model (RASM) with automatic initialization.

The presence of surrounding artifacts and noise in the original pelvic CT images make bone segmentation a challenging task. Therefore, preprocessing is performed to remove the surrounding artifacts (e.g., CT table, cables, hands, and lower extremities) present in the original image. This is the first step in segmentation. The preprocessing is carried out using blob analysis. Later, high-frequency speckle noise is removed from the images using a 2D Gaussian filter. The image is then enhanced to emphasize the features of interest, that is, pelvic bones. This enhancement is done using brightness contrast stretching. Later, the bone edges are detected using Canny edge detection technique. However, some weak edges may remain unconnected, and as such, morphological operations are applied to remove spurious edges and subedge connections and removal.

The obtained preliminary segmentation results are then used to detect the best matching template using a shape matching algorithm [23]. This helps with the automation of the segmentation process and therefore contributes to minimizing human errors during the diagnostic process. 100 bone templates are created from the Visible Human Project dataset manually. These templates are then compared to each CT slice in order to determine the best-matched template. Determining best-matched template enables the application of corresponding training shape models of each best-matched template to the preprocessed image during bone segmentation phase.

The last step in the segmentation process is the extraction of pelvic bones. Standard active shape model (ASM) is one of the popular techniques that is generally used for bone segmentation. Standard ASM uses training images labeled with landmark points to generate statistical shape and intensity-level models of a desired object. The shape model can be iteratively deformed to locate the object in a test image [24]. The landmarks are points selected by an expert for the bone region in each registered image during the training phase. The pelvic bones in each original training image have different sizes, rotation angles, and locations which may lead to unstable and unreliable shape models for inaccurate bone segmentation. In addition, standard ASM is highly sensitive to initialization and requires an initial position to be correctly assigned to the training model in order to detect a target object in the image. The algorithm then attempts to fit the shape model to the object. If the shape model is not accurately placed, the standard ASM may fail to detect the target object accurately.

**Figure 3:**
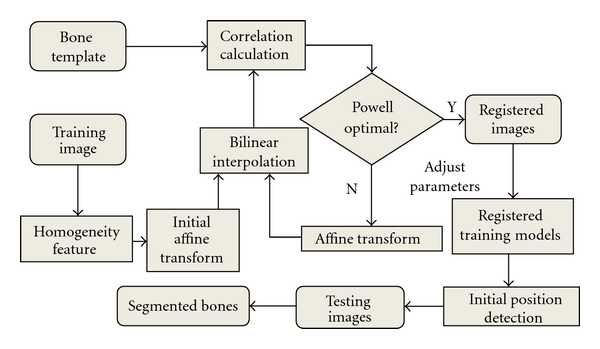
RASM Algorithm.

In order to overcome these shortcomings, a new image registration algorithm, that is, registered active shape model (RASM), is developed using enhanced homogeneity feature extraction [15], correlation coefficient calculation for similarity measure, affine transformation, and Powell algorithm application [25]. This algorithm, that is, RASM, is developed to create a set of more robust training models which will result in more accurate segmentation. This includes two stages: training stage in which registered training models are created and testing stage which includes automatic initialization.  Figure 3. provides the flowchart for the RASM algorithm. After the creation of training models, segmentation is performed on the test images. As mentioned earlier, manual initialization may fail to segment the targeted objects accurately. Hence, an automated hierarchical initialization algorithm is used in the study. The proposed initialization process involves image registration, bone extraction, and edge detection to automatically and sequentially place the training models of each individual object for the test images to extract the bone from the background.

### 2.2. Fracture Detection in Pelvic CT Images

After bone segmentation, a multistage process is used for fracture detection in pelvic CT scans. Fracture detection of pelvic bones includes several steps. First, pelvic bone segmentation is conducted using the proposed RASM algorithm, as described in Section 2.1. The extracted bone boundaries are utilized to create a series of adaptive windows. Later, 2D stationary wavelet transform (SWT) is applied to each window to test the contour discontinuities in each window using boundary tracing. If there is a contour discontinuity in a window, then it is considered as a potential bone fracture.

#### 2.2.1. Adaptive Window Creation

Discontinuities around the bone boundary help identify the presence of fracture. Therefore, a detailed view of bone boundary is required through the formation of windows around the bone whose sizes are adaptively adjusted to include the bone borders. Creation of these adaptive windows around the bone boundary will facilitate the process of identifying the discontinuities. In this study, a systematic method is proposed to form adaptive windows around the bone boundary to include and detect possible discontinuities associated with fractures. The appearance of bone fractures in a pelvic CT scan depends on the injury severity. Major fractures are usually visible, while minor fractures may not severely distort the edge of the bone; instead, they may appear as dual edges or a single subedge that is slightly blurred compared to the neighboring edges. Therefore, it is important to refine the blurred boundary of each bone in order to achieve accurate fracture detection. The refinement is done using a wavelet transform which is later described in the following subsections. However, due to local intensity variations, it may be difficult to achieve practical and desirable results by applying wavelet transform to the entire bone structure. Hence, the detected bone boundary is divided into a series of windows. The size and location of each window is determined by the area of the bone and boundary detected using the RASM. This is called adaptive windowing. The adaptive windowing algorithm is explained in detail as follows.

On the basis of the segmentation formed by the RASM algorithm, the landmarks are placed on the boundary of each segmented bone. The windows are created starting from the first segmented pelvic bone region. The adaptive window is created on the basis of each landmark placed on the segmented bone boundary.

Let {(*x*_*p*1_, *y*_*p*1_), (*x*_*p*2_, *y*_*p*2_),…, (*x*_*pl*_, *y*_*pl*_)}, *p* = 1,2,…, *N*, be the coordinates of the landmarks of each bone in the image. *N* is the number of bones, and *l* is the number of landmarks for each pelvic bone. The landmarks are located at the center position (*C*_*p*_, *D*_*p*_) of each window. The area of the window *W*_*l*_ is determined using

(1) $$W_{l}=\frac{A_{l}}{6},$$

where *A*_*p*_ is the area of the corresponding piece of bone, The determined empirical constant 1/6 has been selected to ensure that the size of the window is appropriately selected. The side length of the each leg of the cubicle (square) window is identified using

(2) $$S_{l}=\sqrt{\frac{A_{l}}{6}}.$$

**Figure 4:**
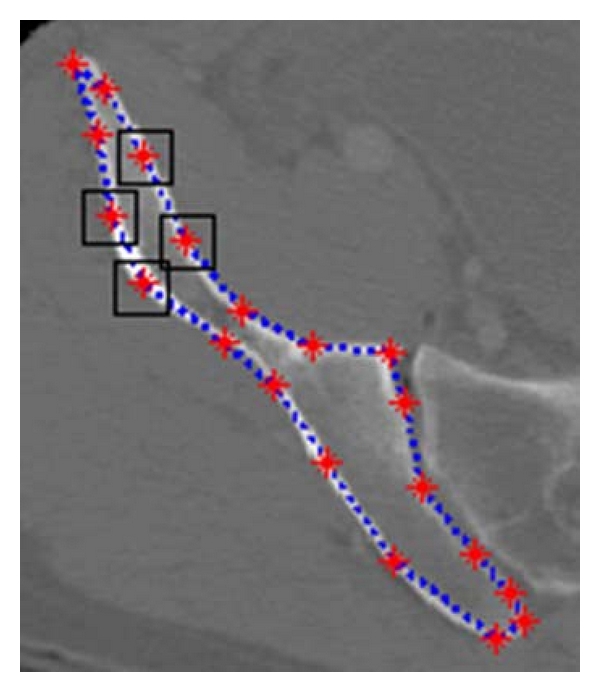
Example windows around the boundary of pelvic bone, positioned according to landmarks.

Since the area of each adaptive window is small, in order to obtain more suitable virtualization effects, each window is scaled to the size of 256 × 256 by applying the bilinear interpolation technique [14]. As shown in Figure 4, sample adaptive windows are created. Each landmark is located at the center of each window.

#### 2.2.2. The 2D Stationary Wavelet Transform

After adaptive windowing, 2D stationary wavelet transform (SWT) is applied on each window in order to refine the blurred boundary of pelvic bone. The classic discrete wavelet transform (DWT) suffers a shortcoming that the DWT of a translated version of a signal/image is not, in general, the translated version of the DWT of the signal/image. To overcome this, SWT is applied in our work, as it is designed to overcome any shift variation [26]. The wavelet transform algorithm is explained as follows.

The wavelet transform decomposes an input signal into different frequency components using a series of filtering operations. A wavelet $\overset{̅}{\varphi_{a}}(t)$ is a function with a zero average

(3) ∫ψ(t)dt=0.

The wavelet generates a family of wavelets by scaling *ψ*(*t*) by *a* and translating it by *θ*:

(4) $$\varphi_{\theta ,a}\left(t\right)=\frac{1}{\sqrt{a}}\varphi \left(\frac{t-\theta }{a}\right).$$

The wavelet transform of a signal *s*(*t*) at time *θ* and scale *a* can be represented as

(5) $$\begin{array}{c} \begin{array}{c} W_{s}\left(\theta ,a\right)=\langle s\left(t\right),\varphi_{\theta ,a}\left(t\right)\rangle \end{array},\\ W_{s}\left(\theta ,a\right)=\overset{+\infty }{\underset{-\infty }{\int }}s\left(t\right)\frac{1}{\sqrt{a}}\varphi^{*}\left(\frac{t-\theta }{a}\right)dt. \end{array}$$

The convolution computes the wavelet transform of the input signal with dilated band-pass filters. Two sets of coefficients are obtained through wavelet transform, one is approximation coefficients, *cA*_*j*_, and the other is detail coefficients, *cD*_*j*_, where *j* is the level of decomposition, including horizontal, vertical, and diagonal coefficients. Decimation makes wavelet transform a shift-variant process. To overcome this, a stationary discrete wavelet transform is used in this study.

**Figure 5:**
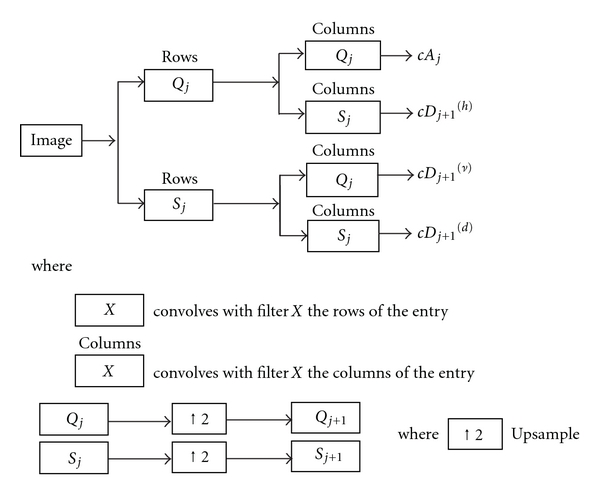
Decomposition steps of 2D SWT.

The scaled window *W* is first decomposed using a 2D Stationary Discrete Wavelet Transform. The classical Discrete Wavelet Transform (DWT) is not a space-invariant transform. The SWT is an algorithm which does not decimate the coefficients at every level of decomposition [26]. The filters at level *i* are upsampled versions of those at level (*i* − 1). As with the 2D DWT, decomposition outputs approximation, horizontal, vertical, and diagonal coefficients. In this application, three levels of decomposition are applied to window *W* using the Haar wavelet. The level 3 detail coefficients, *cD*_*j*+1_^(*h*)^, *cD*_*j*+1_^(*ν*)^, and *cD*_*j*+1_^(*d*)^, are then extracted and used to reconstruct detail arrays *D*_*h*_, *D*_*v*_, and *D*_*d*_ of horizontal, vertical, and diagonal coefficients.  Figure 5 represents decomposition of 2D SWT.

The accuracy and running speed of the SWT algorithm are compared when extracting the upsampled coefficients separately at 1st, 2nd, 3rd, and 4th levels. The algorithm runs on the computer with 2.80 GHz Intel(R) Core(TM) i7 processor, 64-bit Operating System, 6.0 GB memory. For each CT slice, it takes approximately 0.15 seconds more for the 2nd level of stationary wavelet decomposition than the 1st level decomposition. While the 3rd level of decomposition is only 0.1 second slower than the 2nd level of decomposition in terms of running speed, more noise is filtered out, and edges are clearer in the 3rd level of decomposition compared to other two levels; this improves the accuracy of the fracture detection algorithm. Going to the 4th level adds another 0.15 second of additional delay while not adding much to the filtering performance. Hence, in order to achieve a suitable balance between the running speed and accuracy, the 3rd level of SWT is used in this work.

#### 2.2.3. Masking

The next step in the fracture detection is to create a binary version of the chosen detail array *W*_*b*_ from the wavelet transform. This binary version not only contains the pelvic bone contour, but also includes other redundant and unnecessary edges. A mask is formed to filter these redundant edges out. The mask *W*_*m*_ is formed by converting the smoothed window to a binary image using Otsu's threshold [27]. The threshold is computed to minimize the intraclass variance, defined as a weighted sum of variances of two classes, black and white pixels.

(6) $$\sigma_{w}^{2}\left(t\right)=w_{1}\left(t\right)\sigma_{1}^{2}\left(t\right)+w_{2}\left(t\right)\sigma_{2}^{2}\left(t\right).$$

Weights *w*_*i*_ are probabilities of the two classes separated by a threshold *t* and *σ*_*i*_^2^ variances of these classes. Minimizing the intraclass variance is the same as maximizing interclass variance

(7) $$\sigma_{b}^{2}\left(t\right)=\sigma^{2}-\sigma_{w}^{2}\left(t\right)=w_{1}\left(t\right)w_{2}\left(t\right){\left[\mu_{1}\left(t\right)-\mu_{2}\left(t\right)\right]}^{2},$$

where *w*_*i*_ are probabilities of the two classes and *μ*_*i*_ is the class mean.

The contour is then extracted from the binary image. The unwanted edges are removed from the binary image to create an edge window. Later, a precise edge window *W*_*e*_ is obtained by removing the extra edges in the image using the pelvic bone contour and the mask. The process is defined as a combination of *W*_*b*_ and *W*_*m*_. This edge window is used for the boundary tracing as described in next step

(8) $$W_{e}=W_{b}\times W_{m}.$$

#### 2.2.4. Boundary Tracing

After masking, the last and final step in fracture detection is the detection of discontinuities. This is achieved by tracing the extracted bone edges. Small artifacts surrounding the extracted bone edges may interfere with the boundary tracing. Therefore, these artifacts must be removed. These are removed by applying morphologic opening to all the objects in the image with area below a specific threshold, which is predefined as 1% of the window area in the testing step. The remaining edges are then traced using the 8-neighborhood of each pixel and are returned as a matrix of pixel positions. The traced edges represent the pelvic bone contours. The window will therefore contain a single continuous boundary if there is no fracture. In the presence of fracture, multiple boundaries are present in the window, depending on the type and severity of fracture.

## 3. Results and Discussion

### 3.1. Dataset

The dataset has been obtained from the Virginia Commonwealth University Medical Center. Data have been collected from twelve patients with traumatic pelvic injuries. Forty-five to seventy-five images are collected from each patient. Axial CT images with five millimeter slice thickness are used for the study. Images collected from five patients are used for training, and the other seven patients' images are used for testing. For fracture detection, a total of 12 patients are used, out of which 8 patients exhibit small to very severe bone fractures.

### 3.2. Results of Bone Segmentation

**Figure 6:**
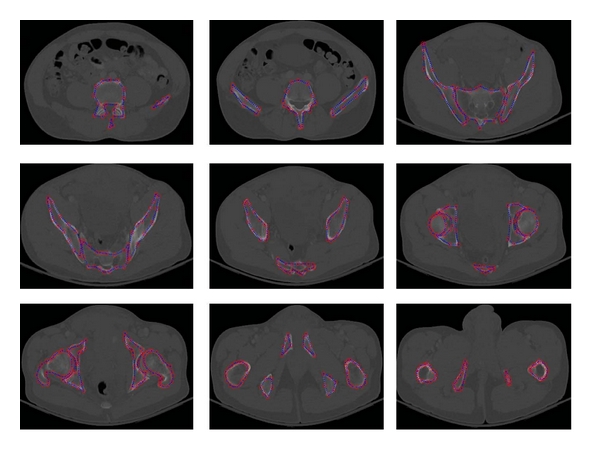
Example of pelvic bone segmentation results via RASM.

**Figure 7:**
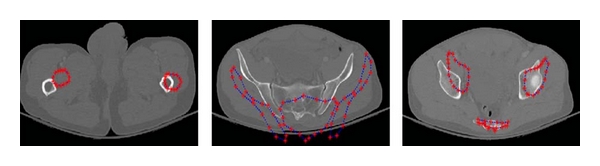
Example results of pelvic bone segmentation via standard ASM without initialization.

Figure 6 shows a sample segmentation of pelvic bones using RASM.  Figure 7 shows the compared results of pelvic bone segmentation via standard ASM without initialization. The main reason of inaccurate bone segmentation is that the initial positions of training models are not correctly assigned. As given in [8], total segmentation accuracy for both good and acceptable classes is 95.77%. These results were evaluated by expert radiologist as ground truth for assessment.

### 3.3. Results of Fracture Detection

**Figure 8:**
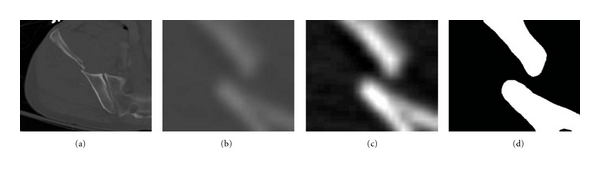
Example of a detected broken boundary of pelvic bone, which may indicate a fracture.

**Figure 9:**
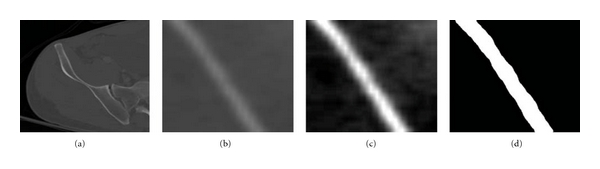
Example of a detected nonbroken boundary of pelvic bone, which may indicate no fracture.

**Figure 10:**
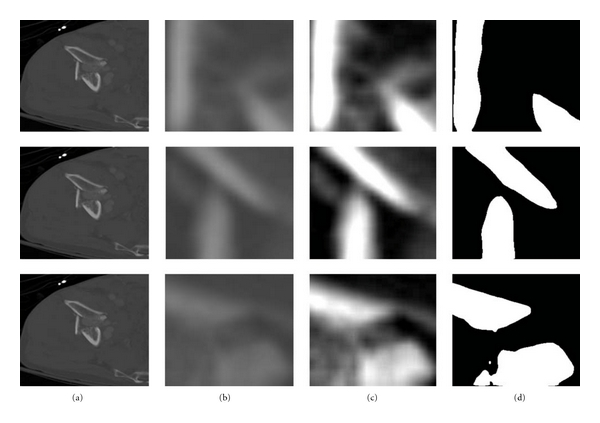
Example of a detected broken boundary of pelvic bone, which may indicate three fractures.

Figures 8 through 10 show the results obtained at various stages of fracture detection. In these figures, (a) is the original image, (b) is the extracted adaptive window after being scaled, and (c) is the enhanced window after brightness contrast stretching. This is done for better visualization effect. And, (d) shows the final fracture detection results. In Figure 8, the patient suffers from a minor fracture in right iliac wing. Figure 8(d) indicates the fracture detected in the right iliac wing.  Figure 9 is the “no fracture” case. The result in Figure 9(d) shows that the bone appears smooth with no fracture.  Figure 10 illustrates a patient with a very severe fracture in the right ilium bone. Fractures are detected from the windows of this bone region. Example of detected fractures shown in Figure 10(d) indicates fractures in three different regions of the right ilium bone. These results are evaluated by an expert radiologist and are considered acceptable. For 8% of the cases, the method was unable to capture the fracture. The few cases that the algorithm gave false alarms in fracture detection may be either due to the algorithm needing further refinement or other factors such as the poor quality of these particular CT images.

The results show that the method can successfully detect bone fracture.  Table 1 presents the performance of the method detecting fractures. The proposed method is highly sensitive to the discontinuities present in the bone and is capable of detecting fractures.

### 3.4. Discussion

The results were validated on the basis of the assessment and evaluation made by radiologists on the CT scans in the above mentioned database. As shown in the results, the designed algorithm is able to detect the fractures relatively accurately. Using the proposed algorithm, fractured bone may be further highlighted in the processed images; this could help the radiologists better analyze the scans and increase the chances of capturing the fractures. Additionally, as it can be seen in the results, our designed method may help quantify the fracture separation distance and the angle between the broken bone pieces as well as other quantitative assessment of the fractures, which may not be easily accessible and measurable through visual inspection. The designed algorithm provides these clues and recommendations on the fracture detection in an automated fashion and with relatively high speed (the processing time is less than one second for each slice). This helps physicians reduce the decision-making and diagnostic time, which is highly important for traumatic pelvic injuries.

## 4. Conclusion and Future Work

This paper presents a method for detecting fractures in pelvic bones using automated bone segmentation, adaptive windowing, boundary tracing, and 2D stationary wavelet Transform while including anatomical information. The results show that the proposed method is capable of detecting fractures in pelvic bones accurately. Automated fracture detection, once verified with more data, will be an important component of a larger modular system to extract features from CT images for a computer-assisted decision-making system. Future work will focus on the quantitative measurement of fracture on the basis of a larger dataset, for example, horizontal displacement, as well as the determination of fracture type.

## Figures and Tables

**Table 1:** Performance of pelvic bone fracture detection.

Statistical Results	Accuracy	Sensitivity	Specificity
Rate %	91.9821	93.3333	89.2617
